# Internists’ dilemmas in their interactions with chronically ill patients; A comparison of their interaction strategies and dilemmas in two different medical contexts

**DOI:** 10.1371/journal.pone.0194133

**Published:** 2018-05-30

**Authors:** Nicolien M. H. Kromme, Kees T. B. Ahaus, Reinold O. B. Gans, Harry B. M. van de Wiel

**Affiliations:** 1 University of Groningen, University Medical Center Groningen, SectorA Chronic and Vascular Disease, Groningen, The Netherlands; 2 University of Groningen, Faculty of Economics and Business, Centre of Expertise Healthwise, Groningen, The Netherlands; 3 University of Groningen, University Medical Center Groningen, Groningen, The Netherlands; 4 University of Groningen, University Medical Center Groningen, Department of Internal Medicine, Groningen, The Netherlands; 5 University of Groningen, University Medical Center Groningen, Wenckebach Institute, Groningen, The Netherlands; Albano Hospital, ITALY

## Abstract

**Background:**

Internists appear to define productive interactions, key concept of the Chronic Care Model, as goal-directed, catalyzed by achieving rapport, and depending on the medical context: i.e. medically explained symptoms (MES) or medically unexplained symptoms (MUS).

**Objective:**

To explore internists’ interaction strategy discourses in the context of MES and MUS.

**Methods:**

We interviewed twenty internists working in a Dutch academic hospital, identified relevant text fragments in the interview transcripts and analyzed the data based on a discourse analysis approach.

**Results:**

We identified four interaction strategy discourses: relating, structuring, exploring, and influencing. Each was characterized by a dilemma: relating by ‘creating nearness versus keeping distance’; structuring by ‘giving space versus taking control’; exploring by ‘asking for physical versus psychosocial causes’; and influencing by ‘taking responsibility versus accepting a patient’s choice. The balance sought in these dilemmas depended on whether the patient’s symptoms were medically explained or unexplained (MES or MUS). Towards MUS the internists tended to maintain greater distance, take more control, ask more cautiously questions related to psychosocial causes, and take less responsibility for shared decision making.

**Discussion and conclusions:**

Adopting a basic distinction between MES and MUS, the internists in our study appeared to seek a different balance in each of four rather fundamental clinical dilemmas. Balancing these dilemmas seemed more difficult regarding MUS where the internists seemed more distancing and controlling, and tended to draw on their medical expertise. Moving in this direction is counterproductive and in contradiction to guidelines which emphasize that MUS patients warrant emotional support requiring a shift towards interpersonal, empathic communication.

## Introduction

In the Chronic Care Model ‘productive interactions’ between an ‘informed, activated’ patient and a ‘prepared, proactive’ physician/practice team’ are linked to positive health outcomes[1,2]. To further clarify this crucial but not yet clearly defined concept, we found that internists define a productive interaction with chronically ill patients as one that is goal directed and catalyzed by achieving rapport[3]. Moreover, the significance of the goals appeared to depend on the nature of a patient’s medical complaint in terms of whether it reflected medically explained symptoms (MES) or medically unexplained symptoms (MUS).

It is generally acknowledged that the extent to which physicians’ interactions are goal-directed depends on the medical context[4,5]. However, there is no research investigating physicians’ interaction strategies in the context of productive interactions comparing MES to MUS.

This distinction between MES and MUS is important because each domain has its own clinical challenges. In the context of MES a global issue is the coordination of care of the complex chronically ill throughout the acute and chronic phases of their illness while safeguarding a holistic approach[6]. In the chronic stage a major interaction challenge is how to engage (complex) chronically ill patients in self-care and how to provide doctors with the skills to motivate patients to adopt self-care[7–9]. Activated patients and physicians who are skilled in supporting patients to adopt self-care are supposed to have productive interactions leading to collaborative management of a patients’ illness[10, 11] and finally to better health outcomes[12–14]. Strategies belonging to motivational interviewing and five specific strategies (concerning information giving and shared decision-making) are propagated as effective strategies to improve collaborative and self-management[15,16]. Few studies, however, explored physicians’ communication strategies in practice as for instance in the case of noncompliant patients[17].

Regarding patients with MUS, a main issue seems to be their frequent use of health care resources [18, 19] as well as the challenge how to interact with these so called ‘hateful’ patients[20,21]. Other studies point to the high degree of (psychiatric) co-morbidity among MUS patients with negative effects on their quality of life[22–25]. Although it is argued that a therapeutic relationship is important for the health outcomes of MUS as well for MES patients[26,27], physicians seem to take patients with MUS less serious than other patients with chronic conditions because they do not have a clear ‘disease’ status[28,29]. Effective interaction strategies regarding patients with MUS, however, appear to matter in primary care as well as in medical specialty care[30–32]. Concerning primary care an explorative study found that community physicians as well as patients considered the following strategies as being effective: exploring causes of symptoms with tests and referrals, attentive listening, validating a patient’s complaints, providing clear explanations of the symptoms, and demonstrating commitment by the physician[30]. Effective strategies identified in a literature overview on medical specialty care (with e.g. positive effect on patients’ anxiety, coping, satisfaction and healthcare use) are perceiving patients' expectations correctly, properly explaining symptoms and giving positive feedback[32]. (See for literature search and brief review: S1 Text.).

The aim of the present study is to explore how internists describe their interaction strategies to achieve productive interaction goals and how their discourse is affected by the medical context: i.e. MES or MUS. Because of the growing number of the complex chronically ill, with medically explained as well as unexplained symptoms asking frequently for specialist care, we were specifically interested in the interaction strategies of internists. The breadth and depth of their profession enables them to deal with the complex problems of the chronically ill at a generalist as well as specialist level and thus may reduce the need to engage other medical specialists[33]. The research questions are:

○ What interaction strategies do internists express for achieving productive interaction goals with chronically ill patients?

- How do the contexts of MES and MUS affect the interaction strategy discourse of internists?

## Methods

### Design

As part of an explorative study on what constitutes a productive interaction of internists with chronically ill (out-)patients, this qualitative study was designed to explore internists’ interaction strategies for achieving productive interaction goals in two medical contexts.

We conducted interviews with twenty internists and coded the transcripts based on a constructivist approach of grounded theory[34]. From this dataset we selected the code categories relevant to our research questions and investigated the selected data by using a discourse analysis approach[35]. Discourse analysis (DA) offers the opportunity to explore human strategic behavior which is context bound and often implicit or covert, and cannot be derived from straightforward questions or observations. The basic premise in DA is that how people make sense of what others say is based on the situational context and their interpretation of what is said in this context. In addition, people’s expressions are characterized and influenced by their practices and their identity, which is based on their membership of social groups[35]. Transposing these ideas to our study, we first argue that, during consultations, internists will verbally construct interaction strategies which, as such, are at least partly and often implicitly determined by what ‘the internist group’ practices, believes, and values. Second, internists will expect others to interpret their sayings and doings in the context of their medical practice and their professional identity. An interaction strategy we define as (implicitly) planned communicative interpersonal actions to achieve an interaction goal.

In defining talk or ‘discourse’, we follow Gee[35] who defines discourse as a characteristic way of saying, doing, and being ([35] pp.2-5) albeit without clear boundaries ([35] pp.36-39). We focused on the participants’ natural speech about clinical practice to investigate the language the participants characteristically use and which reflects their constructed reality[36]. This approach enabled us to identify the strategy discourse employed by the participants and to derive implicit meanings within their language, such as ones related to medical context. We furthermore draw on cognitive anthropological theory[37] that considers a person’s discourse as representing collective understandings that are based on the shared experiences and identity of people who are members of the same social group. (See for a summary of the main concepts used in this perspective on DA: S2 Text.)

### Study sample and data collection

Twenty internists participated in the period from October 2011 to April 2012. These internists worked in the Department of Internal Medicine at a University Medical Centre in the Netherlands. We included internists who had responded directly to our request for participants plus others that we individually approached based on our sampling requirements. To create variation within the studied group, we selected respondents of four different Internal Medicine subspecialties, who also differed in gender and age as shown in Table 1. Variation is needed if one is to generate valid categories, themes, and/or patterns from the data collected and to be able to compare subgroups within the overall group[34]. The selected respondents from General Internal Medicine and Elderly Medicine/Geriatrics can be characterized by their focus on acute care, diagnostics, and short follow up and are seen as generalists. The selected respondents from Endocrinology and Nephrology also have a diagnostic task but are more focused on chronic care (treatment and longitudinal follow up) and are seen as subspecialists. In the Netherlands both generalists and subspecialists, are trained and work as internists. Having satisfied the relevant saturation criterion[38], we stopped seeking additional interviewees when new issues no longer arose during the interviews.

**Table 1:** Characteristics of the participants.

Characteristics of the internists	Number included (% of total staff)
Sub-discipline:	
Generalists:	10 (26%)
Elderly Medicine/Geriatrics	4 (11%)
General Internal Medicine	6 (16%)
Subspecialists:	10 (26%)
Endocrinology	5 (13%)
Nephrology	5 (13%)
Gender:	
Men	12 (32%)
Women	8 (21%)
Age (and gender division):	
34–41 year (5 women, 4 men)	9 (24%)
45–61 year (3 women, 8 men)	11 (29%)

The first author started the semi-structured interviews with an explanation of the Chronic Care Model and posing some general questions about the interviewee’s background and interest in medicine. The topics were introduced in a flexible way and the interviews were held as natural conversations[39]. When the participants talked about the issues they encountered during their interactions with patients the interviewer probed further and/or paraphrased the interviewee’s responses to check that she had understood them [23]. New and relevant issues that arose were included in subsequent interviews. The interviews lasted 44–108 minutes and were fully transcribed with the verbal informed consent of the interviewees. We used the Atlas.ti software program (version 7.5.10) to support the analysis of the transcripts and the data management.

### Data analysis

**Fig 1:**
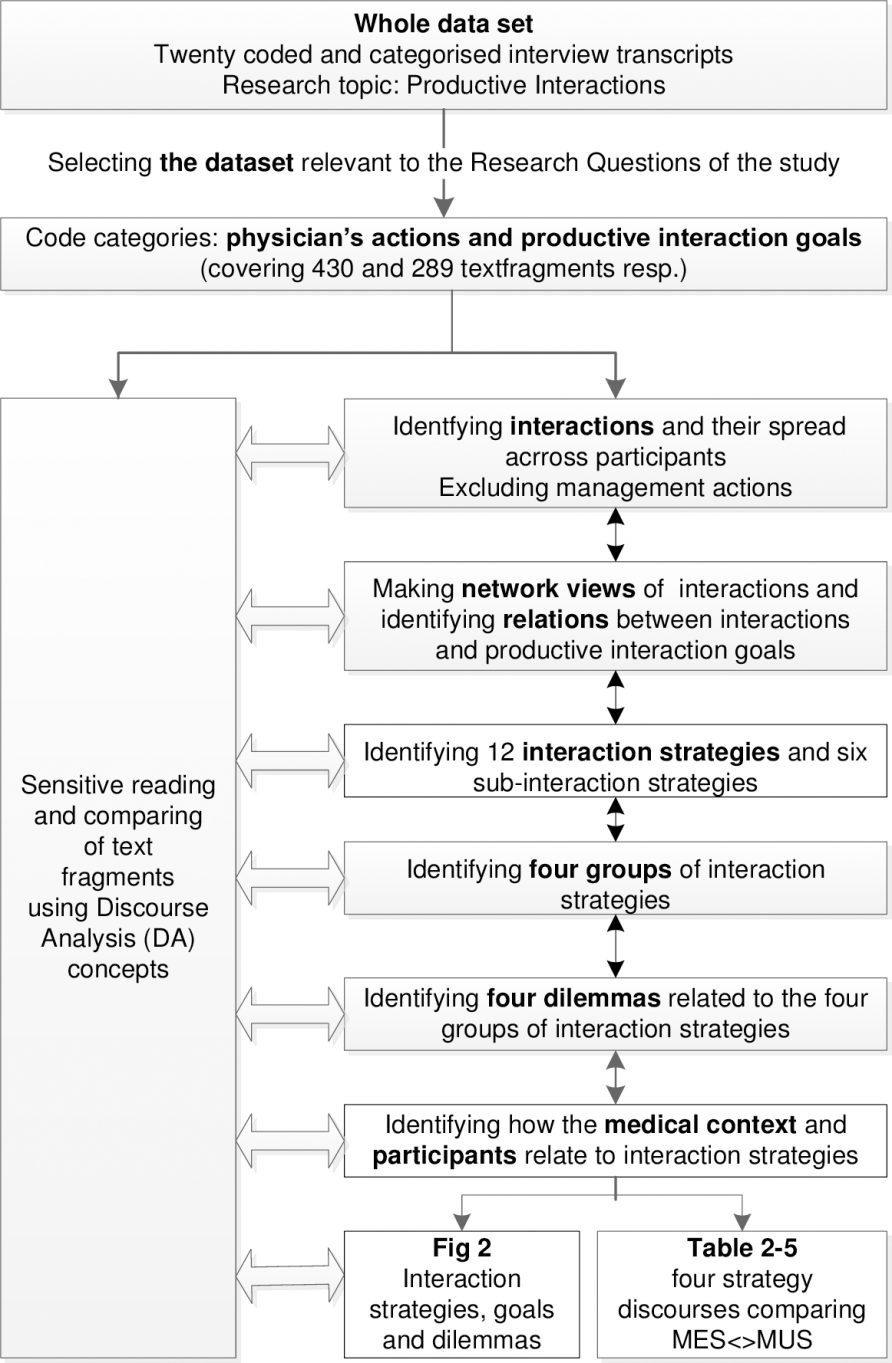
Analysis process.

We selected the category ‘physicians’ actions’ from the coded dataset to form the specific dataset for this study (see Fig 1). We read the included text fragments in this dataset carefully in order to detect meaningful pieces of text that were relevant to the current research topic. Given codes were checked and compared across all the interviews for variability and consistency within the research group consisting of the four authors (NK, KA, RG, HvdW). As we were sensitive to the relationship between interaction strategies and productive interaction goals we discussed text fragments and codes, and solved discussions, to form a final code category of ‘interaction strategies’. In order to identify patterns in how the participants expressed their interactions in the medical context of MES and MUS, we selected Discourse Analysis (DA) concepts or ‘tools’ that suited our study[40]. We investigated how the participants used language to give ‘significance’ to their interactions and practices. To determine the influence of the context we were sensitive to what they did not say but what they implicitly expected the listener to ‘fill in’. Further, we considered whether a participant’s ‘saying’ was ‘framed’ in a broader context and looked at how the participants used ‘typical language’ or referred to ‘cultural models’ and/or to their ‘identity’ (see S2 Text.).

**Fig 2:**
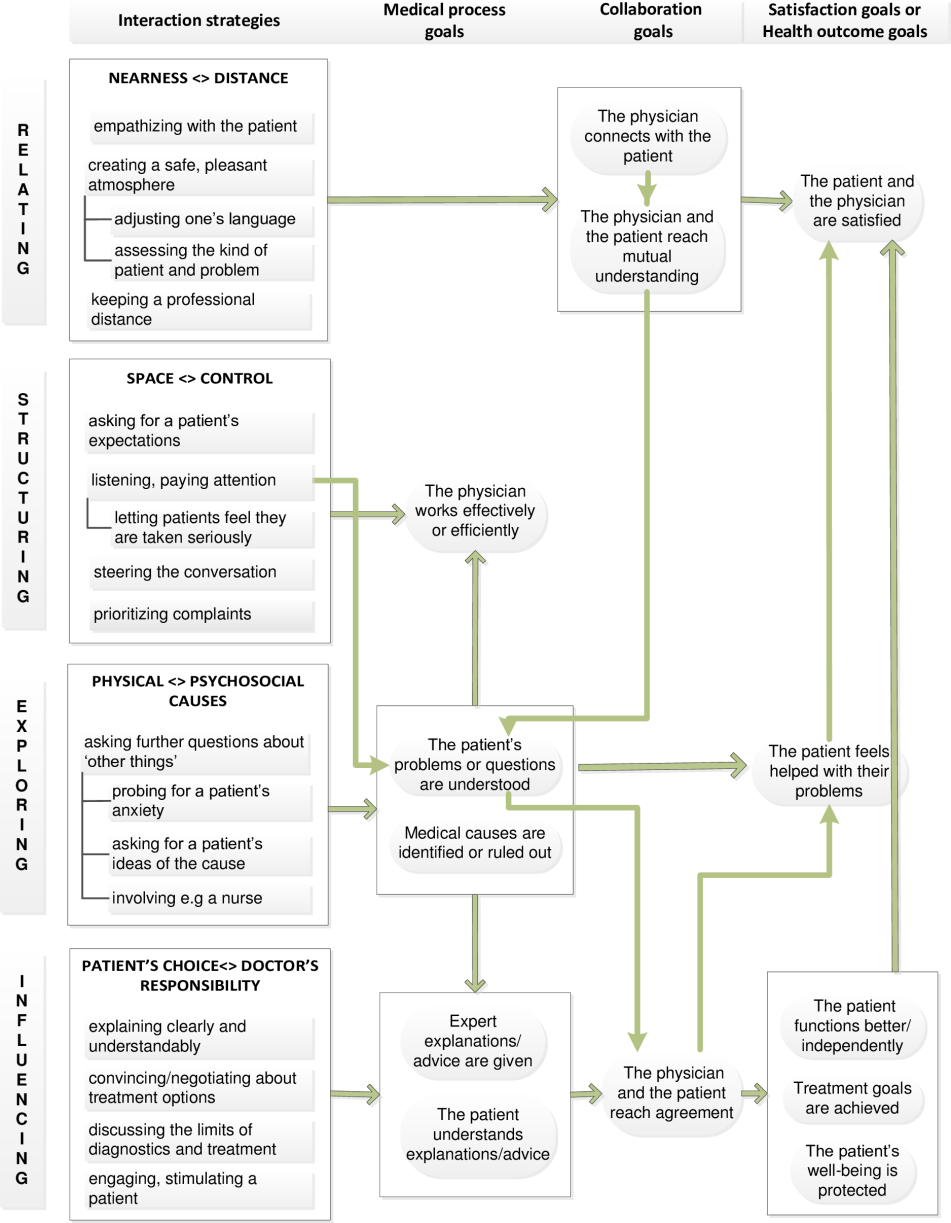
Relationship between physicians’ interaction strategies and productive interaction goals. In total eighteen interaction strategies could be identified and clustered into four main interaction categories (capitals left of a block) each referring to a dilemma (capitals within a block). Three interaction strategies consist of one or more subcategories (column 1). Green arrows show how the interaction strategies are connected to medical process and collaboration goals (column 2) and to health outcome and patient & physician satisfaction goals (column3).

This inductive, iterative analysis resulted into the identification of four groups of interaction strategies that were connected to different productive interaction goals (see Fig 2) and next into four correspondent strategy discourses that each expressed a different dilemma. Moreover, the impact of these dilemmas differed when faced with MUS and MUS. To validate our findings, we systematically verified the data by comparing the content and the use of language within the different fragments with each other. In addition, we extensively discussed the identification and categorization of the interaction strategies as well as the dilemmas we derived. These discussions resolved any discrepancies and led to the final labeling and clustering into four interaction strategy discourses and four corresponding dilemmas.

### Ethical approval

We submitted the overall study proposal for approval to the Committee for Medical Ethics of the University Medical Center Groningen. They decided the study did not require ethical approval under the Dutch ‘Medical Research Involving Human Subjects Act’ (WMO). Initially, we requested oral consent for reasons of establishing an open, informal interview climate and building a trustful relationship during the interviews which was important to our method. At the start of the interview we again explained the broad aim of the study and why tape recording and transcribing the interviews was necessary. We promised to store and use the data confidentially and obtained consent with the procedures of all selected participants. Finally, we asked and obtained written informed consent of all participants to publish their quotes in this manuscript and in the supporting information. Our research adheres to the standards as described in the Standards for Reporting Qualitative Research (SRQR)[41].

## Results

We identified four interaction strategy discourses: relating, structuring, exploring, and influencing. Each interaction strategy discourse could be characterized by a dilemma: creating nearness versus distance; giving space versus taking control; asking further about physical versus psychosocial causes; and taking responsibility versus accepting a patient’s choice (see Fig 2.). The findings for each dilemma are specified into a set of five items that draw our attention to how the four discourses referred to the context of MES or MUS: a. interaction goals, b. interaction strategies, c. interaction issues d. doctor’s image of the patient and e. perceived role of the doctor (Tables 2–5).

**Table 2:** Relating: Creating nearness <> keeping distance.

	Relating: Creating nearness <> keeping distance	
	MES	MUS
**Interaction goals**	Connecting and mutual understanding	Connecting and mutual understanding
**Interaction strategies**	Empathizing with the patient	
	Creating a pleasant, safe atmosphere	Creating a pleasant, safe atmosphere
	○ Adapting one’s language	○ Assessing the kind of patient and problem
	Keeping a professional distance	Keeping a professional distance
**Issues**	Familiarity in long term relationships	Distrust towards the physician
**Doctor’s image of the Patient**	P is seriously ill, at risk for complications, needs to be cared for	P is unlikely to have a serious illness
**Perceived role of the Doctor**	D is expert, caregiver, counselor/coach; has an informal or formal style	D is expert; sometimes counselor, acts businesslike

**Table 3:** Structuring: Giving space <> taking control.

	Structuring: Giving space <> taking control	
	MES	MUS
**Interaction goals**	Working effectively	Working efficiently
**Interaction strategies**	Listening, paying attention	Asking for a patient’s expectations
	Steering the conversation	Steering the conversation
	Prioritizing complaints	Prioritizing complaints
**Issues**	Frustration because of lack of time to be able to listen to everything	Waste of time experiencing by the physician
**Doctor’s image of the Patient**	P needs time to tell their story	P pours out a whole set of problems
		P needs to feel to be taken seriously
**Perceived role of the Doctor**	D pays attention but has to work effectively which is difficult because of the time constraints	D reserves time to listen but has to work efficiently

**Table 4:** Exploring: Focusing on physical <> psychosocial causes.

	Exploring: Asking further about physical <> psychosocial causes	
	MES	MUS
**Interaction goals**	Patients’ problems are understood	Medical causes are ruled out
**Interaction strategies**	Asking further about ‘other things’	Asking further about ‘other things’
	○ probing a patient’s anxiety	○ probing a patient’s anxiety
	○ involving e.g. a nurse	○ asking about a patient’s ideas
**Issues**	Having (not) enough time to explore the patient as a whole	Uncertainty (is really nothing wrong?) Cautiousness (because patients easily feel stigmatized)
**Doctor’s image of the Patient**	P may be anxious; needs reassurance	P may be anxious; needs reassurance
	P does not always tell everything	P conveys endless lists of ailments
	P’s partner or bringing lists is helpful	P’s partner or bringing lists is not helpful
**Perceived role of the Doctor**	D is a biomedical expert who pays more or less attention to the patient as a whole	D is a biomedical expert looking for (physical) and psychosocial causes

**Table 5:** Influencing: Taking responsibility <> accepting the patients’ choice.

	Influencing: taking responsibility <> accepting a patient’s choice	
	MES	MUS
**Goals**	Patient understands explanations/advice. Reaching agreement	Patient understands explanations (that their complaints are not worrisome)
**Interaction strategies**	Explaining clearly	Explaining clearly
	Convincing and negotiating	Convincing and negotiating
	Discussing the limits of *treatment*	Discussing the limits of *diagnostics*
	Engaging and stimulating the patient	
**Issues**	Frustration (P does not recognize blood pressure or abnormal lab results as a serious problem and does not hear or understand the advice)	Feeling burdened by persistent demands; giving in by the doctor
**Image of the patient**	P may be low (health) literate	P is demanding
	P is entitled to make their own choice	P pressurizes the doctor
**Perceived role of the doctor**	D feels responsible for directing/guiding the patient in decision-making	D responsible for ‘first do no harm’ by preventing superfluous diagnostic tests

### Relating: Creating nearness versus keeping distance

#### MES

While the interaction strategy ‘empathizing with the patient’ was often associated with somewhat spontaneously emerging positive feelings and/or identification with the patient, the strategies ‘creating a safe, pleasant atmosphere’ and ‘adjusting one’s language’ were more explicitly aimed at connecting with the patient ‘*by allowing people to feel comfortable and safe and preventing them from feeling like just any other patient*.*’*. For instance by remembering their personal characteristics and background: *‘when I have heard that her husband has died* … *) I try to remember that*.*’*.

In order to give the patient the idea that the doctor knows what it means to be ill, some at times disclosed a little of their own experiences as a patient or as a family member of a patient. *‘*Creating a pleasant and safe atmosphere’ referred also to connecting with the patient by socializing: ‘*not just be businesslike*, *but also try to make contact*, *such as by making a joke*.*’*.

In addition, several participants expressed an informal style: *‘I am comfortable with being on a first name basis with all my patients*. Others, however, preferred a more formal and distant style:

‘I keep a certain distance …) one I am comfortable with …) not too close …) otherwise it impedes the medical relationship and it always feels slightly manipulative.’ (P12, subspecialist)

Some also expressed that familiarity cloud become a problem in long-term relationships.

Irrespective of their style, however, it generally seemed important to build a trustworthy relationship with chronically ill patients in order to let them experience a sense of safety, trust, security and expertise.

#### MUS

The participating physicians appeared to sense and asses this kind of patient and problem immediately and mentioned it could be more difficult to connect with MUS patients because they more often appeared to distrust the physician from the start. In such situations, empathizing and creating a pleasant atmosphere occurred less frequently. MUS patients were also seen as demanding and in combination with psychiatric disorders as manipulative. We often heard that in these situations the participants would respond in a distant, businesslike manner.

### Structuring: Giving space versus taking control

#### MES

The general line of reasoning appeared to be that if you allow patients at the start to tell their story, and/or let them talk for a few minutes, you will receive a lot of information, may be even the diagnosis. However, the participants’ statements about listening and giving a patient space at the start of a consultation were often directly followed by references to steering and structuring: *‘At first I let them speak and then I pose questions and try to bring structure to it*. Prioritizing the problems followed after the patient told their story: *‘Usually I pose a very general question …) then I let them talk for a while and then I try to bring it back to the main issues’*. In the case of elderly co-morbid patients, the participants talked relatively more about prioritizing problems.

The ‘steering’ strategy appeared to be related to the goal ‘working effectively and efficiently’, and time schedules:

‘Sometimes it takes strong steering to keep control and then you stop things [i.e. discussing issues] you think are not relevant; it [the consultation] has to be completed within 15 minutes.’(P22, subspecialist)

In addition, participants often expressed frustration that they could not give their patients enough time to tell their story.

#### MUS

The strategy of ‘asking for a patient’s expectations’ was heard in the event of patients claiming chronic fatigue or when providing a second opinion: ‘*then I ask first*, *what do you expect from me*, *why are you here*?*’* Some, however, said they would adjust a chronic fatigue patient’s expectations immediately: ‘*to explain that it is very unlikely that there is a physical explanation for a problem that has existed for so long*.’ Others, felt that it could be distracting for patients to be told immediately that their problem likely has no physical cause.

Although the schedules allowed the generalists more time in the case of MUS, several participants emphasized the need to steer the conversation with MUS patients:

‘I think I give most patients plenty of space to tell me about their problem, but I steer much more with people who come to the clinic with a whole set of unrecognized symptoms.’(P8, generalist)

Moreover, they frequently used exaggerations during the interview to underline the necessity of steering in such circumstances:

‘I do not have the **entire** morning, you have to do everything within the allotted time slot …) and one does not have **three** hours for that …)so when people pour out an **enormously** elaborate story you will steer that a bit.’ (P9, generalist)

They also noted that ‘letting patients feel they are taken seriously’ was in particular important with patients with MUS. Moreover, the expression of this sub-strategy was regularly accompanied by reflecting on the inconvenience of listening to a MUS patient’s issues:

‘Sometimes I ignore it on purpose (pause) …) that is a choice I make, although I feel it is important [for the patient] but sometimes (pause) …) I think I will get very many stories that I do not want at that moment.’ (P7, generalist)

### Exploring: Focusing on physical versus psychosocial causes

#### MES

To achieve the goal that ‘the patient’s problems or questions are understood’, the participants spoke of ‘asking further questions about other things’ that should be addressed. These unspoken ‘other things’ were associated with psychosocial issues or problems, e.g. issues patients were afraid of to raise or with intimacies such as disabilities or loneliness.

In addition several participants spoke of the sub-strategy of ‘probing a patient’s anxiety’ about having cancer:

‘otherwise you will never reach that shared decision making if you do not know …) what they are actually afraid of; thus, when I think someone is afraid of having cancer, I try to bring this to the surface.’ (P14, generalist)

Although, participants sometimes asked directly about a patient’s worries about having cancer in order to reassure them, they often probed more carefully: *‘oh are there other things [you would like to bring up]*?*’* Some also mentioned that one should have achieved a certain degree of intimacy before probing at hidden things such as loneliness or depression.

Several participants said it was important to investigate a patient ‘as a whole person’ although they acknowledged that this could easily be forgotten while being busy. Others, however, thought that they should restrict themselves to the medical task at hand, or that they were not the appropriate person to explore the social or mental problems of their patients.

#### MUS

When the participants discussed patients they recognized as having MUS, they regularly referred to them as ‘those’ or ‘these’ people. The participants not only seemed more distant but also were more cautious when it came to raise ‘other issues’ in order to prevent MUS patients’ feeling they were being stigmatized:

‘Those people come to you to rule out something medical and if you move too quickly to discussing their background [to assess psychosocial functioning], they lose faith in you as a doctor because they think: ‘that doctor thinks it is all in the head’.’ (P3, generalist)

Cautiousness also concerned the participants’ awareness of the need ‘*not to overlook a serious condition’* and some participants expressed the fear of a claim against them. Given that a doctor cannot be completely sure that nothing is wrong, and patients need reassurance, some participants expressed the view that they wanted to give patients, who asked for a second opinion because of a diagnosis MUS: ‘*the feeling that this time everything really*, *really has been done [to exclude a medical explanation]*.*’*

When we asked the participants whether they explored ‘a patient’s own ideas of the cause of their illness’ some replied that only when they had no real idea themselves, they would ask for the patient’s own ideas. Others would not ask this in order to prevent they should have to listen to an ‘information overload’. Most participants associated MUS patients with bringing endless lists of ailments that had a negative effect on the interaction between doctor and patient. Bringing a partner for support (often emphasizing the degree of suffering) was not seen as helpful.

### Influencing: Taking responsibility versus accepting the patients’ choice

#### MES

‘Explaining clearly’, e.g. what the risks and the consequences of a medical condition are, was an often mentioned strategy. Several participants emphasized they liked to explain everything very clearly because:

‘In the end, it is about the patient’s health and therefore they need to be fully informed about …) the pros and cons [of the therapeutic possibilities].’ (P15, generalist)

The participants, however, regularly complained about time pressure and that ‘*things do not always come across’* as they would like:

‘We have half an hour [for a new patient] …) and then having to explain in the last few minutes what it means to have a [.] disease …) that is not possible! I have noticed several times during follow-up appointments that patients …) did not understand at all why they were here and what that meant.’ (P16, subspecialist)

Some therefore emphasized that they would ask whether patients had understood what they had discussed and appreciated it when a patient’s partner was present.

The ‘convincing and negotiating’ strategy referred to balancing the agendas of both parties.

In the event of disagreement about the advice offered, participants found it important to have in the open why a patient was unwilling to follow an advice. If a patient were to say: ‘*No*, *I absolutely do not want this*, *I have had high blood pressure my whole life’*, several participants would accept this as their patient’s choice. When they felt it had (severe) adverse effect on a patient’s health, they e.g. said: ‘*then I apply more pressure*, *although in the end of course the patient still decides*.*’* While they were inclined to accept a patient’s choice, they also felt responsible for guiding patients with severe medical conditions: ‘*Yes*, *a sort of coaching …) but (pause) as a doctor you have to be directive as well*, *because that is what patients often expect from you*.*’* When it came to elderly patients, several participants expressed the view that they as physicians were best able to assess the risks and consequences of the medical condition for the patient: ‘*I think I am the one who …) although I usually do not put it like this*, *knows best at this moment*.*’* Trying to convince a patient, however, was not always seen as productive, for example when a patient was upset.

In addition to negotiating, ‘discussing the limits’ of a treatment plan (e.g. side-effects of medication) some participants mentioned they asked the patient:

‘How far do you actually want to go [with treatment]? …If you get a stroke, do you want everything possible done?’ (P14, generalist)

Finally, ‘engaging or stimulating’ patients to achieve a health outcome through changing lifestyle was seen as important but not often as very effective. Some offered the opinion that engaging patients in e.g. real-time glucose monitoring, could result in ‘practicing together’[42].This, however, required (already) engaged patients and could lead to time-consuming and thus more expensive care.

#### MUS

Some participants emphasized that one should immediately ‘explain openly and clearly’ the diagnostic possibilities and limitations to avoid giving MUS patients false expectations. Others mentioned that they discussed the need for diagnostic testing with MUS patients, especially with young patients who had searched the internet. ‘*Apparently …) they and their GP …) need reassurance that still nothing is wrong’* or regarding second opinions: *‘you have to deliver more which means further diagnostic tests …) as well as more talking*.*’*

The participants’ main issue seemed to be that while MES patients may not recognize high blood pressure and/or abnormal lab results as a serious problem, MUS patients describe many ailments that are generally not worrisome from a physician’s perspective. Hence, in MUS cases, they felt responsible for limiting unnecessary or harmful diagnostics. However, they regularly felt patients remain demanding.

‘The most difficult is the demanding patient …) who wants certain investigations to be done …) That is more troublesome—then discussions really arise. And it is really important for the physician to know the root cause of their demands …). This is actually the most important reason for my appointments running late.’ (P6, generalist)

However, if they became tired of persistent MUS patients, they may gave in.

## Discussion

This is the first study that explores the interaction strategies that internists adopt to achieve productive interaction goals in the medical contexts of MES and MUS. We identified four interaction strategy discourses and determined how these were related to productive interaction goals. We also derived four dilemmas for which the internists came to different solutions for MES and MUS.

### Strategic dilemmas

‘Creating nearness versus distance’ refers to a classical paradox that a Dutch psychiatrist described as: ‘maximal approach while keeping distance[43]. The internists in our study seemed to balance between involvement and distance. They applied relating strategies in order to reach common ground but some also reflected on being (too) close or (too) involved, implicitly referring to the view (of Osler[44]) that being emotionally involved hampers a calm judgment and may lead to an excessive burden for the physician[45]. Too much distance, however, and a physician fails to develop a therapeutic relationship with a patient and does not elicit the appropriate information. The participants also found that distrust and feeling manipulated affected the relationship negatively and associated distrust more often with MUS patients. The literature supports this finding as the most negative form of communication in general[46,47] and in particular with MUS[48].

‘Giving space versus taking control’ is a well-known dilemma that is addressed in medical education(49) and has evoked reflections about the ‘art of medicine’[50]. The internists in our study generally emphasized the importance of listening to patients and giving patients space to tell their story. Nevertheless, they often simultaneously felt the need to steer because of time pressure. In the case of MUS they emphasized in particular the ‘letting patients feel they are taken seriously’ strategy[28] while they simultaneously felt the conflicting need to ‘control’ MUS patients who were listing endless problems and/or demanding. This also refers to the dilemma between a physician’s limited time and emotional energy, and need to invest in probing clues, which is much more time consuming in MUS cases [51].

‘Asking further about medical versus psychosocial causes’ reflects a disease versus illness focus that is evident in the literature[28,49,52] and relates to the discussion about the need for a biopsychosocial approach[53,54]. Under time pressure, however, the wholeness principle seemed difficult to enact. Notably, the participants often talked about psychosocial issues as ‘other (non-medical) things’ that were ‘hidden beneath the surface’, and they had to seek out. Despite this, the participants would generally not ask patients explicitly for their expectations and/or ideas about the cause of their problems. This contradicts the general importance attached to the elucidation of a patient's ideas, concerns, and expectations in the literature[55] and especially the explanatory models of symptoms[52,56]. Being uncertain about missing a somatic cause in the case of MUS and not really being interested in the views and ideas of the patient, reflects the difficulties doctors experience when diagnosing and treating MUS patients, and may cause cognitive bias[56].

‘Taking responsibility versus accepting a patient’s choice’ refers to the division of power in the patient-doctor relationship[57,58]. While differences between patient and doctor were framed in terms of having different agendas and accepting a patient’s choice, we also regularly heard that ‘the doctor knows best’. The internists considered it was often difficult to involve patients in decision making because patients seemed unable to oversee the risks of or how to cope with their disease. The internists therefore felt the need to take responsibility for decision making. Moreover, this is what patients often expect of physicians[59]. However, while the participants framed a patient’s choice positively in MES cases, they associated it negatively in MUS cases with demanding patients and medical shopping. The literature indicates that when GPs perceive patients as demanding more investigations, patients will receive more[60]. Although the internists in our study seemed somewhat uncertain they also were reluctant to order more investigations and found it more difficult to accept a MUS patient’s demands (choices). This may explain why they seemed less inclined to a process of shared decision making with MUS.

### MES<>MUS

**Fig 3:**
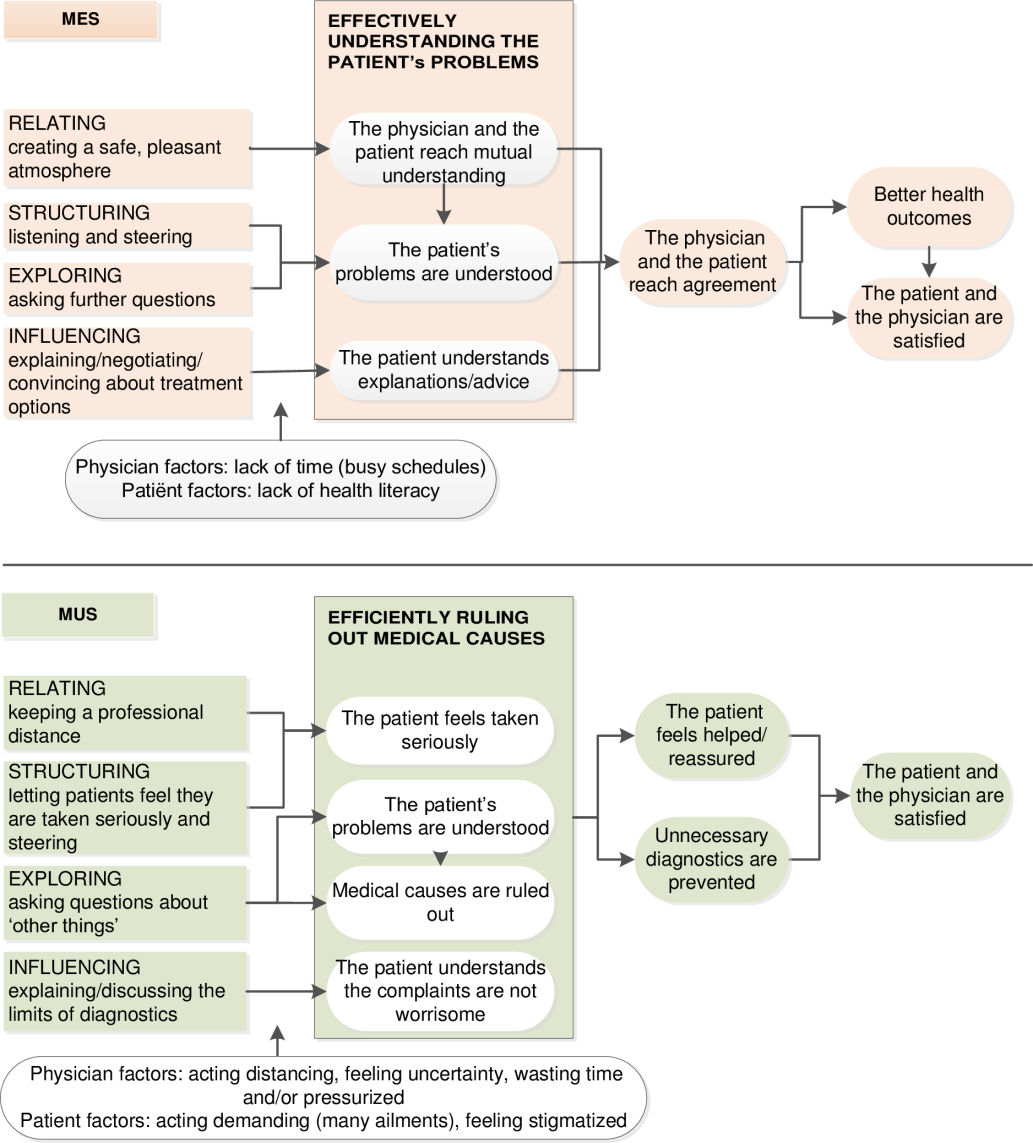
Physician’s interaction strategies to reach productive interaction goals: MES compared to MUS. In the context of Medically Explained Symptoms (MES) physician interaction strategies focus on relating, structuring and influencing (explaining and convincing) while the literature indicates that the focus should shift towards the strategy of activating patients. In the context of Medically Unexplained symptoms(MUS physician interaction strategies focus on structuring, exploring and influencing; while the literature states that MUS patients need relating strategies.

The dilemmas we identified demonstrate that the internists were balancing their behavior and reflected, largely implicit, on what was the right thing to do (see the ethics of Aristotle in Box 1) in both MES and MUS contexts. Balancing, however, seemed more difficult when faced with MUS compared to MES. In Fig 3 we modeled the reasoning of the participants about their interaction strategies in order to reach productive interaction goals comparing the context of MES to MUS.

**Box 1. Ethics of Aristotle**

Aristotle was the first philosopher to address the goal directedness of human behavior. In his Ethica Nicomachea[70], he promotes lifelong learning based on virtues in order to become effective in one’s striving to be virtuous or excellent. He tried to make his pupils aware that virtues can help someone choose the appropriate behavior: for example not to be too brave but also not to act like a coward. The consequence is a lifelong struggle in search of the right balance, which is different in each situation and interaction with each person. Aristotle distinguishes two kinds of virtues: virtues of intellect (involving knowledge, skills, etc.) and of character (involving continuous acting and coping with emotions that finally form one’s character). It took reflection on one’s actions, including discussions in one’s community, and a whole life to integrate experiences into excellent personal qualities and to become ‘whole as a human’.

The strategic reasoning of the participants in the context of MES we called: ‘effectively understanding the patient’s problems’. During the acute as well as the chronic phases of a MES patient’s illness, the participants basically relied on a establishing a good relationship[61] to reach mutual understanding and agreement and finally better health outcomes. This focus on relating strategies is consistent with studies that point to the positive effects of a therapeutic relationship[62–64] but contradicts research findings that focusing on patient activation in the chronic phase is a more effective strategy[9,14]. The internists, however, did not often engage patients although they seemed to be influenced by the societal discourse about patient choice. They primarily explained, convinced and/or negotiated, and generally felt time constraints. Their spoken comments sounded largely paternalistic[65] but also resembled (shared) negotiated decision making based on medical expertise which may be seen as moving in the direction of patient-centeredness[66,67].

In the context of MUS we called the participants’ strategic reasoning ‘efficiently ruling out medical causes’. Compared to MES the participants’ seemed to maintain a greater distance, take more control, ask more cautiously questions related to possible psychosocial causes and take less responsibility for the MUS patient’s health. Issues associated with the participants’ more negative understandings of MUS were distrustful and demanding patients bringing endless lists, as well as the feeling of uncertainty and wasting time. They also seemed to believe that MUS patients were more inclined to pressure them for a kind of medical treatment. Despite this belief an empirical study found that MUS patients in GP practices did not seek more medical interventions nor more reassurance than other patients; rather they wanted more emotional support from their doctor than did others[68]. Guidelines confirm that a therapeutic relationship (interpersonal nearness) is important for the health outcome of MUS patients and state that MUS warrants a shift towards interpersonal communication[26,68]. The internists’ MUS discourse, however, indicates that they found it difficult to establish a trusting relationship with MUS patients and often responded automatically with distancing while focusing on ruling out medical causes based on their medical expertise. It is also possible that MUS patients, because they are referred to specialists, often expect more or other diagnostic testing as well as a kind of medical intervention. In addition the expectations and behavior of both patients and physicians could be based on their collective understandings of the patient-doctor relationship and medical system [22,66,69].

### Limitations

Although we found general strategies and dilemmas which we suspect would be recognized in other settings, our sample, and in particular our generalist and subspecialist divisions, may not be representative of other practice settings. Another point is that the generalists in our sample spoke more frequently and were (in some cases) more outspoken about MUS while the subspecialists spoke more often about MES as a consequence of their different patient mixture in daily practice. Nevertheless, we could verify in the data that the dilemmas were found within all subgroups as well as how they were affected by the MES and MUS context. The widespread relevance of the general dilemmas we found with different impact on two medical contexts, however, should not be seen as implying that there are no other differences between practices or doctors. Differences between the participants’ discourses also seemed to be affected by their age, gender, and/or individual coping strategies. For example, female participants expressed more often nearness-creating strategies and emphasized they really wanted to listen and were interested in the patient as a whole. Male participants, contrastingly, more often articulated they would respond distantly (authoritatively or businesslike) to demanding or manipulative patients. They also seemed to be more focused on working efficiently and more often expressed the view that ‘asking further about other things’ was not their task. As such, male discourses tended to be more biomedical centered and female discourses more person centered[71], a distinction that was particularly evident when it came to dealing with MUS. These characteristics, styles, or beliefs need to be further investigated.

## Conclusions

In this study, we showed that internists transform the concept of productive interaction into concrete goal directed strategic interaction behaviors that depend on the medical context. Adopting a basic distinction between patients with MES and MUS, they appeared to seek a different balance in each of four rather fundamental clinical dilemmas. With patients with MUS, the internists sought to keep more often a greater distance, take more control, were more cautious over delving into psychosocial causes, and were less eager to take responsibility for shared decision-making than they were with MES patients. Guidelines state this is counterproductive. Research indicates that MUS patients warrant emotional support which requires a shift towards to interpersonal, empathic communication. The internists, however, automatically seem to turn into the opposite direction: keeping distance while drawing on their medical expertise. Because building a therapeutic relationship with MUS patients does not appear to be common practice, although research indicates this is an effective interaction strategy, our findings may have implications for medical educators and policy makers. Implementation of guidelines in current training in clinical practice, apparently, lags behind.

## Supporting information

S1 Text Brief literature review. (PDF)

S2 Text Concepts used in discourse analysis. (PDF)

S3 Text Quotes of the participants. (PDF)

S1 Table Spread of interaction strategies among subgroups of participants. (PDF)
