# Supplementary material for: Internists’ dilemmas in their interactions with chronically ill patients; A comparison of their interaction strategies and dilemmas in two different medical contexts
Source: PLoS One. 2018 May 30;13(5):e0194133. doi: 10.1371/journal.pone.0194133 (PMC5976145; doi:10.1371/journal.pone.0194133)
Supplement: S1 Text — (PDF) [file pone.0194133.s001.pdf]

Manuscript: Internists’ dilemmas in their interactions with chronically ill patients; a comparison of their interaction strategies in two different medical contexts

Supplemental file 1. Brief literature review.

Updated search (2017, October 28)
To support our claim that we did not found records comparing MES to MUS we updated our initial search in 2016 February 10. This resulted in including two more records for full inspection.

Search strategy

EBSCOhost (Medline, SocIndex and Psychinfo) (62 records)
Search terms: TI (communciation or interaction) AND TI strategies AND AB (physician or clinician). Refinement to Language: English

Embase (58 records)
Search terms: (communication:ti OR interaction:ti) AND strategies:ti AND ('physician'/exp OR physician OR 'clinician'/exp OR clinician) AND [english]/lim

Scopus (141 records)
Search terms: TITLE (*communication* OR *interaction*) AND TITLE (*strategies*) AND TITLE-ABS-KEY (*physician* OR *clinician*) AND Refinement to [LIMIT-TO (SUBJAREA , *"MEDI"*) OR LIMIT-TO (SUBJAREA , *"SOCI"*) OR LIMIT-TO (SUBJAREA , *"HEAL"*) OR LIMIT-TO (SUBJAREA , *"ARTS"*) OR LIMIT-TO (SUBJAREA , *"PSYC"*) AND (LIMIT-TO (LANGUAGE , *"English"*))]

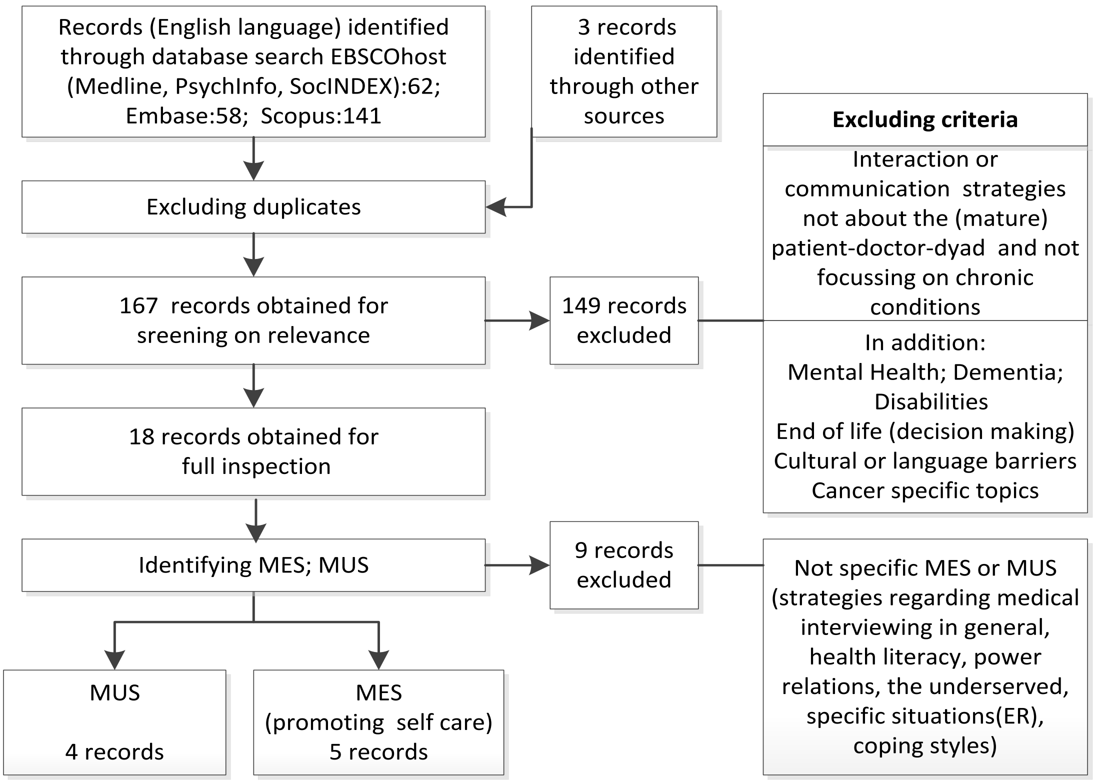

Fig 4. Outline of the identification process

Manuscript: Internists' dilemmas in their interactions with chronically ill patients; a comparison of their interaction strategies in two different medical contexts

Summary Medically Explained Symptoms (MES) concerning promoting self care

- Robins and Wolf (1998)(1) discuss the use of the theory of Brown and Levinson about politeness and strategic language in therapeutic encounters where physicians and patients negotiate about validity and severity of a patient's illness as well as lifestyle changes. Conclusion the theory of Brown and Levinson may help physicians to avoid some communication difficulties.
- Boxer and Snyder (2009)(2) propagate five interlocking strategies for information giving and shared decision making to assist physicians and other caregivers with the collaborative model ('the new paradigm') in order to promote self care: 1. set a shared agenda; 2. ask ,tell, ask; 3. assess readiness to change; 4. set self management goals and 5. close the loop.
- Moir et al. (2009)(3) discuss that short interventions, motivational interviewing (styles and strategies), the catastrophe model and the stages of change are useful frameworks to promote behavioral change of patients. In short consultations, the skilful use of empathy is important for establishing rapport, in order to influence behavioral change and optimize self care.
- Zuiches (2013)(4) explored noncompliance, depression assessment and communication strategies in out-patient cardiology. Most cardiologist considered noncompliance a common source of difficult patient interactions. Instead of altering their communication most cardiologists kept repeating their lifestyle and medication advice. Some reported interest in changing their communication to foster patient trust and motivation.
- Bonner et al. (2014) (5) demonstrate how GPs use three different communication strategies ('positive, scare tactics and indirect') to address the issue of Cardio Vascular Risk. Absolute risk played a different role in the use of each strategy. Strategies depended on GPs' perception of patients' risk, motivation and anxiety. It was suggested to provide GPs with alternative ways of explaining absolute risk in order to achieve different communication aims.

Summary Medically Unexplained (Physical) Symptoms (MUS)

- Anderson et al. (2008)(6) explored management (and communication) strategies of community physicians. Physicians as well as patients considered e.g. the following strategies as effective: exploring causes of symptoms with tests and referrals, attentive listening, validating a patient's complaints, providing clear explanations of the symptoms, and demonstrating commitment by physician and patient.
- Weiland et al. (2012)(7) provide a literature overview on the influence of medical specialists' communication on outcome and health care use of patients with medically unexplained physical symptoms. Conclusion (8 eligible studies): 'Perceiving patients' expectations correctly enables specialists to influence patients' cognitions, to reduce patients' anxiety and improve patients' satisfaction. Patients report less symptoms and health anxiety when symptoms are properly explained. Positive interaction and feedback reduces use of health care and improves coping.'
- Giroldi et al. (2016)(8) explored communication strategies of GPs. GPs tended to a directive approach towards talkative patients. However, to avoid damaging the relationship they also employed a stepped approach to gather information in a respectful and efficient manner.
- Schuermeyer et al. (2017)(9) discuss a more modern view of challenging patients (primary care as well as hospital care)and present cases provided with guidance on handling them. They conclude: 'understanding common difficult personality types can help doctors plan effective strategies for dealing with each, resulting in more effective communication, less stress, and better health outcomes.'

Manuscript: Internists' dilemmas in their interactions with chronically ill patients; a comparison of their interaction strategies in two different medical contexts

(1) Robins LS, Wolf FM. Confrontation and politeness strategies in physician-patient interactions. Soc Sci Med 1988;27(3):217-221.

(2) Boxer H, Snyder S. Five communication strategies to promote self-management of chronic illness. Fam Pract Manag 2009 Sep-Oct;16(5):12-16.

(3) Moir F, van den Brink R, Fox R, Hawken S. Effective communication strategies to enhance patient self-care. J Prim Health Care 2009 Mar;1(1):67-70.

(4) James Daniel Zuiches. Communicating with Difficult Patients in Outpatient Cardiology: A Qualitative Study Exploring Noncompliance, Depression Assessment, and Communication StrategiesPacific Graduate School of Psychology, Palo Alto University; 2013.

(5) Bonner C, Jansen J, McKinn S, Irwig L, Doust J, Glasziou P, et al. Communicating cardiovascular disease risk: An interview study of General Practitioners' use of absolute risk within tailored communication strategies. BMC Fam Pract 2014;15(1).

(6) Anderson M, Hartz A, Nordin T, Rosenbaum M, Noyes R, James P, et al. Community physicians' strategies for patients with medically unexplained symptoms. Fam Med 2008 Feb;40(2):111-118.

(7) Weiland A, Van de Kraats RE, Blankenstein AH, Van Saase JL, Van der Molen HT, Bramer WM, et al. Encounters between medical specialists and patients with medically unexplained physical symptoms; influences of communication on patient outcomes and use of health care: a literature overview. Perspect Med Educ 2012 Nov;1(4):192-206.

(8) Giroldi E, Veldhuijzen W, Dijkman A, Rozestraten M, Muris J, van der Vleuten C, et al. How to gather information from talkative patients in a respectful and efficient manner: a qualitative study of GPs' communication strategies. Fam Pract 2016 Feb;33(1):100-106.

(9) Schuermeyer IN, Sieke E, Dickstein L, Falcone T, Franco K. Patients with challenging behaviors: Communication strategies. Cleve Clin J Med 2017 Jul;84(7):535-542.
