# Supplementary material for: Internists’ dilemmas in their interactions with chronically ill patients; A comparison of their interaction strategies and dilemmas in two different medical contexts
Source: PLoS One. 2018 May 30;13(5):e0194133. doi: 10.1371/journal.pone.0194133 (PMC5976145; doi:10.1371/journal.pone.0194133)
Supplement: S2 Text — (PDF) [file pone.0194133.s002.pdf]

Manuscript: Internists' dilemmas in their interactions with chronically ill patients; a comparison of their interaction strategies in two different medical contexts

**Supplementary File 2. Concepts used in Discourse Analysis [1]**

**Practices:** how people use language to specify what they practice or do (e.g. sequence of actions) and/or what they try to achieve (e.g. certain goals) in order to be recognized by others.

**Identity:** how people use language to enact their (group) identity or 'being', and relationships with others, by emphasizing their role (e.g. as a real mother) or by comparing their role with others.

**Significance:** how people use language to give certain 'sayings' greater emphasis or relevance than others through using certain words or structures in a certain context (e.g. use of superlatives).

**Context:** e.g., bean means coffee bean in a coffee-making context or soya bean in a food preparation context. People will infer the meaning of 'bean' based on the context. Sometimes people 'frame' their sayings in a broader context by referring to a broader societal discourse, such as about fair trade of coffee beans or of genetic manipulation of soya beans.

**Social language:** how people use typical language (e.g. medical jargon) in certain contexts to define their social position relative to other groups, or to distinguish some groups from others.

**Cultural models or schemas:** understandings that reflect people's relatively stable cognitive structures [2]. Schemas influence how language (e.g. metaphors, typical words) is used to refer to what seems natural to a social group: their usual way of thinking, feeling, believing, valuing, using symbols, tools, etc. This includes social norms about what is viewed as normal, right, good, correct, proper, and/or appropriate. Together these norms and values form a meaning system [3].


1. Gee JP. *How to do discourse analysis: A toolkit.* Second ed. New York: Routledge; 2014.

2. Strauss C, Quinn N. *A cognitive theory of cultural meaning.* Vol 2. 1e ed. New York: Cambridge University Press; 1997.

3. D'Andrade RG. *Culture meaning systems in culture theory, essays on mind, self and emotion.* New York: Cambridge University Press; 1984:88-119.
