# Supplementary material for: Internists’ dilemmas in their interactions with chronically ill patients; A comparison of their interaction strategies and dilemmas in two different medical contexts
Source: PLoS One. 2018 May 30;13(5):e0194133. doi: 10.1371/journal.pone.0194133 (PMC5976145; doi:10.1371/journal.pone.0194133)
Supplement: S3 Text — (PDF) [file pone.0194133.s003.pdf]

Manuscript: Internists' dilemmas in their interactions with chronically ill patients; a comparison of their interaction strategies in two different medical contexts

Supplemental file 3

Quotes of the participants: a minimal set of additional, supporting quotes from the interview transcripts

In presenting the quotes here we follow the structure of the manuscript: interactions strategies and dilemmas in two contexts: Medically Explained Symptoms (MES) and Medically Unexplained Symptoms (MUS).
The quotes (or part of the quotes) used in the manuscript are in *italic* and green color.

Conventions used in the interview transcripts and quotations

The transcripts include everything the respondent and the interviewer said including backchannel words as "ums" and "ahs" of the respondent and the interviewer.
In the quotations, however, we excluded the ums and ahs, removed repetitions, grammatical errors and false starts and used punctuation to improve readability. The use of backchannel words, grammatical errors and punctuation etc. varies between interviews, as it reflects how the respondent speaks.
Quotation marks within a quote are used when the respondent quotes another person (e.g. a patient)
A speaker's longer pause is indicated with the word "pause" round brackets: (pause).
Three dots in round brackets (…) indicate we left less relevant parts of the speakers expressions often including repetitions and/or grammatical errors.
Square brackets are used for the interviewer's comments and sometimes indicate that a specific noun is replaced by a category in order to avoid identification. Square brackets could also indicate the removal of identifying material from the interview with a colon [:]
At the end of the quote we refer with a number to the respondent and to their sub-specialism: a generalist (internist/general internal medicine or internist/elderly medicine) or a subspecialist (internist/ endocrinologist or internist/nephrologist). We did not add the specific sub-specialism, gender or age/experience in order to avoid the identification of the participants.
See for the relation between the quotations and the number and nature of the respondents: Supporting information file 2.

Relating: creating nearness <> distance

MES

Empathizing with the patient

'Yes, it depends on the seriousness of the disease, the complexity of course also (…).Then you are more active (pause), then I am more in touch with patients; letting them come back a few times; quicker telephone calls in between; an appointment to hear how it is going; arrange things faster for them. Thus, I notice that and maybe also for younger patients (…) then I want to have it resolved or treated as quickly as possible or start a trajectory etc. and that absolutely appeals to you. And just a little more guidance, especially when young people lead an active life and have a serious illness, that has a lot more impact, so you'll go into that a bit more.' 7:29 (generalist)

'In addition, I also try to put myself in a patient's shoes even if things do not work out. Suppose I find out that a patient does not take his medication, I raise that and I also ask: 'gee, how come, what are the reasons for that?'.' 17:12 (subspecialist)
'Empathizing with people is generally easy when someone is closer to you, it apparently has limits, you simply cannot empathize with everyone.' 17:26 (subspecialist)

Creating a safe and pleasant atmosphere

To invest from the start in the communication is effective *'by allowing people to feel comfortable and safe and preventing them from feeling like just any other patient'* and also calling [a patient] to tell them the lab results the day after their first visit at the outpatient clinic; it is the feeling that someone is looking after me and cares for me.' 16:3 (subspecialist)

'I sometimes also tell things about myself [:] that I also have members in my family who [:](…) that I know what it [certain treatment] means for a patient, a family.' 16:32 (subspecialist)

'when I have heard that her husband has died or that she had a CVA last year *I try to remember that.' 8:36 (generalist)*

Of course you should *'not just be businesslike, but also try to make contact, such as by making a joke.' 4:34 (subspecialist)*

'I show them…that I am familiar with their situation, know their track record; then I look in their file and say:: 'gosh, you have known Prof. [:] (…) and demonstrate you are committed to them as well (…) *yes, for some people a sense of safety, trustworthiness, security and expertise is important.' 12:19/20 (subspecialist)*

Adjusting one's language

'That your language is understandable for your patient and I hear that sometimes from a patient ... that often medical terms are used or difficult words that people do not understand, then I think that it is very important that we understand each other.' 9:2 (generalist)

'I did not have time to arrange an interpreter and had to talk to her son, but that went very well and I felt that I was really in touch with her (…). Then I will not be sitting behind the desk, but then I will be sitting nearer to her and look attentively at her [in order to make contact] .' 10:46 (generalist)

Keeping a professional distance

'I am comfortable with being on a first name basis with all my patients.' 7:37 (generalist)

'I keep a certain distance (…) one I am comfortable with, that is reasonable in my profession (...) *not too close (…) otherwise it impedes the medical relationship and it always feels slightly manipulative.' 12:11 (subspecialist)*

MUS

Empathizing and Assessing the kind of patient and problem

'One patient evokes more empathy than another (…). Sometimes you do click, and sometimes you feel less of a click. It is a feeling you have as soon as they enter the room, how they look at you, you just see whether they trust you or not (…). Sometimes it will get better but it never becomes entirely good.' 3:19/20 (generalist)

'First it matters how people present their problem and with some people you will click sooner than with other people, then you (…) understand better, are more empathic; with others you know in advance, at the beginning of the conversation, that they will tell you a very long-winded story and that the answer to every question will be that they suffer from it (…). Very soon you know it already.' 5:28 (generalist)

'And people with for example [psychiatric conditions] or with ailments for which it is hard to find a medical cause, are difficult to empathize with.' 8.34 (generalist)

'At the first consultation you already try to feel why people have come [to see you]: have they come in order to be reassured or do they really come because they want an explanation for their complaints.' 9:27 (generalist)

'Some people just appeal to you (…) people who do not complain too much, and have certain characteristics for example, or of a family that you recognize, or values you were brought up with, people who do not complain who just work hard, who also dare to share their feelings.'10:34 (generalist)

Keeping a professional distance

'Sometimes you feel that you need to really behave as a doctor, but that depends on the symptoms. If patients are not easily satisfied with the answer, then you are naturally inclined to be more directive; so if you still cannot figure out together what to do (…). This happens especially in the case of chronic fatigue, abdominal complaints, second opinions where you feel at one point: it is just done, we have checked it, there is no illness. And sometimes you just have to step back a little more.' 7:39 (generalist)

'There are also people with whom I do not have such a good contact (…) or not a special contact, or a businesslike contact. Especially, when you cannot do much about their complaints, then the consultation can be very short although I try to make contact. It very often concerns (…) complaints for which we have no [medical] explanation.' 11:12 (generalist)

'With these people I feel little contact, but then I do what is necessary and then that is (…) that ultimately it remains a fairly businesslike contact.' 11:13 (generalist)

Structuring: giving space versus taking control

MES (reasoning in general)

Listening

'I always begin by asking: 'how are you doing', I do not ask how is your stomach pain, I ask how are you doing, yes (…) approaching them as a person and then they usually tell you what bothers them the most.' 7:19 (generalist)

'That they feel they are being understood and being heard and that they had the opportunity to tell their story.' 9:32 (generalist)

Steering

'At first I let them speak and then I pose questions and try to bring structure to it.' 3:31 (generalist)

'Then I try to steer and try to direct the story [of the patient] to a certain point that I understand: this is what you are saying.' 13:12 (subspecialist)

'Sometimes it takes strong steering to keep control and then you stop things [i.e. discussing issues] you think are not relevant; it [the consultation] has to be completed within 15 minutes.' 22:35 (subspecialist)

Prioritizing

'Usually, I pose a very general question such as what can I do for you, *then I let them talk for a while and then I try to bring it back to the main issues.' 11:30 (generalist)*

MUS

Asking for a patient's expectations

'When people come to me to [for a second or third opinion] (…) after they have already been seen by three doctors, well in such situations I really will not be able to find it [a medical explanation] either, so *then I ask first: 'what do you actually expect from me, why are you here?'* That can really help and creates enormous clarity in the sense (…) that there are problems that cannot be solved.' 20:12 (subspecialist)

'[in a consultation with] a 23-year-old woman with chronic fatigue, I will turn the conversation within 3 minutes to explain that it is very unlikely that there is a physical explanation for a problem that has existed for so long.' 6:5 (generalist)

Listening and steering

'I think I give most patients plenty of space to tell me about their problem but I steer much more with people who come to the clinic with a whole set of unrecognized symptoms.' 8:30 (generalist)

'I do not have the entire morning, you have to do everything within the allotted timeslot (…) and one does not have three hours for that (…) so when people pour out an enormously elaborate story you will try to steer them a bit.' 9:16 (generalist)

'It [whether a partner is present] influences the contact and requires much more control of the conversation, for which, I think, the doctor is responsible.' 11:21 (generalist)

Letting a patient feel they are being taken seriously

'Often there is an underlying fear, fear like there has to be something, something seriously is the matter and you are not taking me seriously (…). The fact that I do not do what they would like me to do, so that means extra examinations.' 6:32 (generalist)

'Also then [patient is referred by the GP] it is important that you first let the patient feel that you take them seriously (…). They need to feel they can tell their story and have been taken seriously, so that is what I always try to do and (…) sometimes you look from a somatic perspective and sometimes from a psychosocial one and that depends on the story, their presentation, but I do believe that people who are referred to a medical specialist have a serious complaint and that includes [those coming for] second opinions.' 7:21 (generalist)

'Sometimes I ignore it on purpose (pause) (…) that is a choice I make, although I feel it is important [for the patient] but sometimes (pause) (…). I think I will get very many stories that I do not want at that moment.' 7:48 (generalist)

'There are people who have much more serious diseases (…) and I will never say this aloud (…) I stress in my consultations (…) that they need to be taken very seriously; that they have a reason for visiting an internist (…).They are bothered with something (…) but I cannot clearly understand that (…) but then I think who am I to judge (…) they are very much troubled, but I cannot help them with that.' 8:20 (generalist)

' You also have to be cautious you do not think; 'here they are again' and that you will also at a given moment no longer take her [partner] too not seriously at a certain moment.' 12:25 (subspecialist)

Exploring: focusing on physical versus psychosocial causes

MES

Asking further question about 'other things'

'It is of course very important, with all the [lifestyle or diet] restrictions people have, that you think: 'how will it be at their home, should there be more help?' If the partner becomes ill, that is of course relevant because (…). They [the partner] is [currently] only just able to keep the system running.' 4:43 (subspecialist)

'With patients who return month after month, you think: they not only have physical problems, there are also other things; of course you notice this quicker when you see people more often.' 17:18 (subspecialist)

Asking about a patient's ideas of the cause' of their illness

'But then [In the case of high blood pressure] I am less focused on that [the patients opinion], compared to people who really have unexplained medical symptoms and I have the feeling they are much more anxious about (…). People who have hypertension do not often think they have cancer (…). Generally, in the case of hypertension, I need to convince them that it is serious because they do not experience this as a serious complaint except when their blood pressure is very high because then they maybe get anxious (…) e.g. about having a stroke (…). That is the flipside actually, that you find an abnormal level that people are not bothered about, while on the other side people often have ailments for which you cannot find a physical cause.' 5:10 (generalist)

Probing a patient's anxiety about having cancer

'Especially the elderly say: 'you are the doctor' (…) but there are some things you will bring to the surface, it is often fear, fear of cancer, especially in elderly patients.' 6:44 (generalist)

'Then people begin with what they think is the reason for their visit and I try to read between the lines (…) and hook onto things I think are important (…) or what may be a hidden agenda (…) (pause) because *otherwise you will never reach that shared decision making if you do not know (…) what they are actually afraid of, thus when I think someone is afraid of having cancer, I try to bring this to the surface 14:11(generalist)*

'Sometimes, It is difficult for a patient to speak their mind. I will always try to ask and sometimes it leads nowhere (…). Then often at the end of a conversation there is a moment to say '*oh are there other things [you would like to bring up]* and yes often that is the moment it will come up. Sometimes there is also a moment at the end to say: 'is there something you are afraid of?' Often I also explain: 'the better I know what you are afraid of, the better I can answer your question.' 14:13 (generalist)

Involving e.g. a nurse

'I found it very productive at that time to do that together with [a nurse]...He [the patient] also told her a lot about the relationship, about the partner (…). She [the nurse] started, then I joined (…). With the three of us a lot of things came to the surface.' 4:32 (subspecialist)

MUS

Asking further questions about 'other things'

'The latter [psychological or social problems] you often see with unexplainable symptoms, fatigue, mood disorders, concentration or memory problems, stomach pain; then I ask more often how are you feeling, are you happy or depressed, can you still enjoy things?' 5:11 (generalist)

'I do pose questions about stress, how it is at home at work (…) and when it concerns a condition where I think the psyche plays a role then I pose more questions and go into it more deeply. But I must not overestimate my role. I restrict myself to the somatic, because that is why the patient has been referred and therefore wants an answer.' 6:13 (generalist)

'Of course, sometimes you have to be very direct, but in the case of people bringing a combined physical and psychological problem (…) you will start with a careful approach asking about other things. People are used to sharing intimate things about their disabilities but to share that you are feeling lonely or down is, I think, quite a step, and then you first need to have reached a certain level of intimacy before you can talk about that and careful formulation then helps.' 10:8 (generalist)

No [not giving space for a patient's own story], partly to protect myself against an enormous information overload and things one just cannot influence from the perspective of a medical relationship.' 19:39 (subspecialist)

Cautiousness

'You have to be careful for *those people come to you to rule out something medical and if you move too quickly to discussing their background [to assess psychosocial functioning], they lose faith in you as a doctor because they think: 'that doctor thinks it is all in the head';* thus you have to be careful not to slip immediately into that role and straight into discussing all that other things.' *3:38 (generalist)*

'With people with fatigue you have to find out a bit what to do (…). You could give them psychological support (…) looking at how they can cope with that further, thus how this fits into their lives. A psychological approach is not always a good option even it may be psychological; that often goes against the grain for many people, like 'but I am not insane'.' 9:28 (generalist)

'Exactly, so you have to be very careful with that [asking at psychosocial things] and try to feel when you can probe this with a patient and when it is better not to do this just yet.' 9:33 (generalist)

Not overlooking a serious condition

'I have the feeling that others [patients] experience the same: and it is primarily the uncertainty, not knowing at all what to do next? And that is often underestimated (…). Recently, I saw a girl, just 18 years old, who is very tired, together with her mother and when I hear the story (…) then I order a lab [test] and think: 'nothing is seriously the matter, and then they come back and are obviously disappointed that I cannot really find anything. Then I'll say: 'come back in six weeks'. One can always be mistaken, you can for instance never rule out a non-Hodgkin's lymphoma immediately.' 3:36/37 (generalist)

'It is especially the reassurance that you will give those people *'the feeling that this time everything really, really has been done' [to exclude a medical explanation].' 3:62 (generalist)*

Asking about a patient's ideas of the cause of their illness

'I do sometimes ask that [a patient's idea of the cause] and especially when I have lost the thread (…) then I ask: 'what do you actually think yourself?' So, sometimes I do that, and especially when I do not have the faintest idea.' 3:31 (generalist)

'Sometimes, I do [(ask a patient's opinion] in the case of vague complaints, chronic fatigue, stomach pain. Some people suffer all kinds of things: concentration problems, sweating, sluggishness (…). It is important to know that, when it is not immediately very clear what the cause is (…). Yes, we still do see many people with unrecognized complaints (…) people with chronic fatigue, that is almost never resolved.' 5:9 (generalist)

'Yes, I often do pose that question: 'what do you think is the matter?', especially in the case of chronic

fatigue it is important to bring that to the surface. So: 'What is wrong? What do you think yourself? What, if I do not find a [physical] cause?'' 6:42 (generalist)

'I try to curb that [patient's ideas about the cause] a bit because then you are endlessly busy because the patient sticks to his own theories and stays in his own world and although you do not want to break down that world, sometimes that world is not really very plausible from a biomedical viewpoint.' 19:8 (subspecialist)

Involving e.g. a nurse (or other discipline)

'It should not be a consultation block full of unexplained symptoms (…). The art of course is to get satisfaction from that (…). Then you have to tap into totally different skills (…). You just have to approach such people very differently. Due to our somatic approach we do not have sufficient skills for this. There is an outpatient clinic where an internist and psychiatrist work together - that absolutely adds value.' 5:38 (generalist)

Influencing: patients' choice versus doctors' responsibility

MES

Explaining clearly

'In the end, it is about the patient's health and therefore they need to be fully informed about (…) the pros and cons' [of the therapeutic possibilities]' and I can try to be as creative as possible, given the [a patient's] preferences, to come up with a solution but sometimes there is no perfect solution.' 15:65 (generalist)

'When a patient says: *'No, I absolutely do not want this [treatment], I have had high blood pressure my whole life and I do not want this' then I will accept that.* Or when they do not take their pills then I will try to explain why [they should] (…) and in the end it is their decision and I can accept that, although I may address it again when we see each other again.' 4:25 (subspecialist)

Things do not always come across

'We have half an hour [for a new patient] (…) and then having to explain in the last few minutes what it means to have a [:] disease (…) that is not possible! I have noticed several times [during follow-up appointments] that patients (…) did not understand at all why they were here and what that meant.' 16:24 (subspecialist)

Convincing and/or negotiating and a patient's choice

I will try to discover why [they do not comply].Very often they fear side effects. I find it important to clarify why some do not [comply] and might say: 'Although it is against my advice, it is of course your choice to follow that advice or not' and sometimes *'then I apply more pressure, although in the end of course the patient still decides.'* 4:71 *(subspecialist)*

'Look, with symptoms like these, I often need to convince them that it is something serious'. 5:10 (generalist)

The patient, of course, often has a completely different agenda, (…). They are suffering from pain in the shoulder and then you think that does not really matter for the outcome (…) but being chronically overhydrated and that [high] phosphate [level] does' 4:75 (subspecialist)

'Some people need …a bit of a pep-talk, like a sports coach gives (…). *Yes, a sort of coaching (…)* it is also a bit more directive than a coach would be *but (pause) as a doctor you have to be directive as well because that is what patients often expect from you.* Anyhow that means they see I take it seriously.' 12:39 (subspecialist)

'Patients can and will make their own choices, but sometimes it is important to put it clearly; '*I think I 'am the one who (…) although I usually do not put it like this, knows best at this moment;'* so I have to explain clearly: 'these are the possibilities and these are the consequences and [ask] do you realize that'.' *10:42 (generalist)*

Discussing the limits of treatment options

'How far do you actually want to go [with treatment]? (…). If you get a stroke, do you want everything possible done?' 14:27 (generalist)

Engaging or stimulating patients

'I say: 'try to work on it yourself' and sometimes I refer them to the dietitian if I think something can be gained from it and yes sometimes I try (…) to find out whether I can motivate the patient to do something.' 13:21 (subspecialist)

'In modern diabetic care (…) you are actually collaboratively searching, *practicing together* on the [blood sugar] regulation. Often it concerns younger people with pumps and some are pregnant and some have continuous glucose sensors (…) but it is not possible with everybody. People have to be capable of working on a PC (…) it cost a lot of time (…) on the other hand it is care that has to be provided.' 22:13/15 (subspecialist)

MUS

Explaining clearly (reassuring)

'[in a consultation with] a 23-year-old woman with chronic fatigue, I turn the conversation within 3 minutes to explain that it is very unlikely that a problem that has existed for so long, has a physical explanation.' 6:5 (generalist)

'When another 25-year-old states: 'I have been tired for ten years', of course you think: 'not another one who probably has nothing serious' (…). *Apparently (…) they and their GP (…) need reassurance that still nothing is wrong.'* 7:28 (generalist)

Discussing the limits of diagnostics and convincing a patient

'And regarding second opinions you have to deliver more which means further diagnostic tests, even though you think nothing will be gained, as well as more talking.' *3:62 (generalist)*

'When we became doctors, we promised we would not harm people; so you do not expose them to radiation when you do not expect anything will be gained from it'. 9:26 (generalist)

'When there really is no good reason, I try to convince them, but in the end if they persist, I sometimes say: 'OK, we will do it, but I really do not see a reason for it.' 5:17 (generalist)

'When it is not clear (…) we have to look further (…) and if it is necessary I will say: 'we need this and that [kind of investigations] to confirm what I or the GP suspect' (…) and that conversation indeed will proceed differently in the case of fatigue.' 6:25 (generalist)

'The most difficult is the demanding patient (…) who wants certain investigations to be done (…) and we as doctors have to deal increasingly with that (…) thus, the claiming patient who wants certain investigations done or who is not satisfied when, at a certain moment, it is said: 'no further investigations will be done; it is now over. *That is more troublesome - then real discussions arise. And then it is really important for the physician to know the root cause of their demands (…).This is actually the most important reason for my appointments running late.' 6:28 (generalist)*
