# Supplementary material for: Internists’ dilemmas in their interactions with chronically ill patients; A comparison of their interaction strategies and dilemmas in two different medical contexts
Source: PLoS One. 2018 May 30;13(5):e0194133. doi: 10.1371/journal.pone.0194133 (PMC5976145; doi:10.1371/journal.pone.0194133)
Supplement: S1 Table — (PDF) [file pone.0194133.s004.pdf]

Manuscript: Internists' dilemmas in their interactions with chronically ill patients; a comparison of their interaction strategies in two different medical contexts

Supplemental file 4

Spread of interaction strategies among participants (generalists compared to subspecialists, male compared to female and young compared to old)

**Interaction strategies codes** divided over four categories	Times a code was used to label a text fragment	Number Subspec: Generalist	Number Male: Female	Number Young: Old
a) **Relating**				
• empathizing with a patient	27	6s:6g	8m:4f	5y:7o
• creating a pleasant, safe atmosphere	29	7s:5g	8m:4f	4y:8o
○ adjusting one's language	16	7s:4g	4m:5f	3y:6o
○ assessing the kind of patient and problem	47	10s:8g	12m:6f	
• keeping a professional distance	20	7s:4g	11m:4f	4y:7o
**Sub Total**	**139**			
b) **Structuring**				
• asking at a patient's expectations	12	3s:5g	4m:4f	3y:3o
• listening, paying attention	42	5s:9g	8m:6f	7y:7o
○ letting patients feel they are taken seriously	20	2s:5g	4m:3f	3y:4o
• steering the conversation	28	7s:9g	9m:7f	7y:9o
• prioritizing complaints	20	2s:8g	6m:4f	6y:4o
**Sub Total**	**122**			
**c) Exploring**				
• asking further at 'other things'	43	7s:10g	11m:6f	9y:9o
○ probing a patient's anxiety	20	6s:9g	8m:7f	8y:7o
○ asking at a patient's ideas of the cause	22	6s:9g	8m:7f	8y:6o
• involving e.g. a nurse (or psychiatric)	23	7s:6g	7m:6f	5y:7o
**Sub Total**	**108**			
d) **Influencing**				
• explaining clearly and understandably	26	6s:6g	7m:5f	6y:6o
• convincing and negotiating	18	8s:3g	8m:3f	3y:8o
• discussing the limits of treatment	25	7s:7g	7m:7f	5y:7o
• activating, stimulating a patient	31	8s:2g	7m:3f	3y:7o
**Sub Total**	**120**			
**Total**	**469**			

The table displays the interaction strategy codes (four categories) and their spread among participants .
In total interaction strategy codes were used 469 times to label 346 text fragments (NB some text fragments were labeled two times with two different codes).
The 1[e] column of values column counts how many text fragments were labeled with an interaction (strategy) code.
The 2[e] column shows the number of subspecialists (of total 10) that expressed a specific strategy versus the number of generalists (of total 10).
The 3[e] column shows the number of male (of total 11) that expressed a specific strategy versus the number of female (of total 8).
The 4e column shows the number of young, i.e. 34-41years old (of total 9) that expressed a specific strategy versus the number of old, i.e.45-61years old (of total11).
